# Editorial: Plant biology for indoor vertical farming: a multi-discipline approach to controlled environment agriculture

**DOI:** 10.3389/fpls.2025.1675562

**Published:** 2025-10-31

**Authors:** Paul P. G. Gauthier, Leo F. M. Marcelis

**Affiliations:** ^1^ The University of Queensland, Brisbane, QLD, Australia; ^2^ Centre for Horticultural Science, Queensland Alliance for Agriculture and Food Innovation, Saint Lucia, QLD, Australia; ^3^ Wageningen University & Research, Wageningen, Netherlands

**Keywords:** vertical farming (VF), controlled environment agriculture (CEA), nutrient use efficiency (NUE), light spectrum management, plant-microbes interaction, nitrogen and phosphorus cycling, hydroponics, precision climate control

One of the most significant challenges of the 21st century is feeding a growing population while minimizing environmental impact amid climate change, water and nutrient scarcity, extreme weather, and biodiversity loss. By 2050, the global population is projected to reach 9–10 billion, 60% of whom will live in regions characterized by limited agricultural output and increased vulnerability to food insecurity as a result of climate stressors (see Tchonkouang et al., 2024, and Marzi et al., 2021, for a review). Meeting the FAO’s projected 70% increase in food production by 2050 (FAO, 2009) will require innovative production systems that go beyond conventional agriculture (Van Dijk et al., 2021). One of these alternative approaches is vertical farming, which can produce food independently of weather or seasons. With its potential for high yields, space efficiency, and resource optimization, vertical farming stands at the forefront of agricultural research and innovation. However, realizing its full potential requires more than just stacking plants indoors and relying on technology to address challenges. Balancing its inherent high energy costs demands an integrated understanding of controlled environments, high-density cropping systems, and plant physiological responses.

This Research Topic of *Frontiers in Plant Science* explores the multidisciplinary approaches necessary to advance indoor vertical farming. It highlights the critical need for integrating research across plant biology, cultivar selection, environmental science, and technological innovations to optimize crop production in controlled, high-density environments.

## Optimizing nutrient dynamics for sustainable crop production in controlled environments

Controlled environment agriculture (CEA), including vertical farming, has transformed food production through hydroponic and aeroponic systems that allow for precise control over nutrient delivery. However, effective nutrient management remains a critical factor for optimizing yield, maintaining crop quality, and achieving environmental sustainability. While the role of macronutrients such as nitrogen (N), phosphorus (P), and potassium (K) is well-established, secondary nutrients such as chloride and micronutrients also play essential roles in plant physiology and crop performance.

Phosphorus, a crucial nutrient for plant growth, can become a limiting factor in CEA systems due to its potential for leaching. Westmoreland and Bugbee demonstrated that excessive phosphorus application to *Cannabis sativa* did not improve yield or quality but rather significantly increased nutrient runoff, thereby raising environmental concerns. Similarly, He et al. investigated nitrogen metabolism under varying light conditions, demonstrating that increased light intensity enhances nitrogen assimilation but also increases nitrate reductase activity and total nitrogen content. Nitrate reductase is known to be a light-dependent enzyme (Deng et al., 1991; Lillo, 1994), and its positive response to increased light intensity confirms its role in modulating nitrogen assimilation in photosynthetic leaves. These findings underscore the necessity of light-optimized fertigation strategies to maintain a balance between photosynthetic efficiency and nutrient use efficiencies.

Microbial solutions offer promising approaches for nutrient recovery and leachate utilization. Tan et al. explored the role of *Trichoderma harzianum* in phosphorus and nitrogen uptake, revealing that its efficacy is highly dependent on light conditions. Under high-light conditions, *Trichoderma* enhances nutrient uptake; however, under low-light conditions, it may shift toward parasitism, competing with the plant for resources (discussed further below). These findings highlight the need for strategic integration of beneficial microbes within hydroponic nutrient management systems to optimize nutrient cycling and minimize inefficiencies.

Chloride, often viewed as a stress factor, is also critical for photosynthesis, osmotic balance, and ion homeostasis (White and Broadley, 2001; Raven, 2017, but see also Li et al., 2017). Fitzner et al. investigated chloride accumulation in halophytes and found that light regimes and salinity levels significantly influence chloride uptake and stress responses. Their results suggest that improper chloride management in hydroponic solutions can disrupt plant water relations and nutrient balance in salt-sensitive crops. Given the complexities of nutrient interactions in high-density CEA systems, adaptive fertigation models that integrate real-time nutrient monitoring (Lim et al., 2024, but see also Ahamed et al., 2025), advanced mass balance (Langenfeld et al., 2022), or microbial cycling strategies are necessary for optimizing plant growth and sustainability.

## Harnessing light strategies to enhance photosynthetic efficiency and crop performance

Light management is a fundamental component of vertical farming, influencing plant growth, development, and resource use efficiency. The spectral composition, intensity, and duration of light exposure regulate key physiological processes, including photosynthesis, photomorphogenesis, and secondary metabolite production (Kaiser et al., 2024). While red and blue light have traditionally been optimized for plant growth, recent studies emphasize the roles of far-red light (Demotes-Mainard et al., 2016; Zhen et al., 2022; Kelly and Runkle, 2024; Shomali et al., 2025), green wavelengths (Smith et al., 2017; Liu and Van Iersel, 2021; Chen et al., 2024; Paradiso et al., 2025), and upward lighting strategies (Zhang et al., 2015; Yamori et al., 2021) in modulating plant responses.

The spectral composition of light affects both photosynthetic efficiency and plant morphology. Van de Velde et al. demonstrated that far-red supplementation enhanced the light-use efficiency in butterhead lettuce by promoting leaf expansion and photon capture rather than directly increasing photosynthesis. However, excessive far-red exposure led to reduced chlorophyll content and increased stress markers, indicating the need for precise control of spectral tuning. Similarly, Saito and Goto investigated upward lighting strategies, showing that redistributing light within dense canopies improves net photosynthetic rates and carbon assimilation efficiency. These findings highlight the potential of spectral and spatial light optimization in mitigating shading effects in high-density cultivation.

Light intensity and duration also impact nutrient assimilation and overall crop productivity. He et al. examined the effects of different light intensities and durations on *Portulaca oleracea*, demonstrating that increased light exposure enhances nitrogen metabolism, root and shoot biomass accumulation, and nitrate reductase activity. However, continuous light exposure negatively affected photosynthetic efficiency, emphasizing the need for optimized photoperiod management. Fitzner et al. investigated the effects of different spectra on halophytes and found that light quality significantly influenced pigment accumulation, stress tolerance, and overall metabolic stability under saline conditions.

These findings underscore the necessity of dynamic, responsive lighting systems in vertical farming, as highlighted by Abedi et al. and Kaiser et al. (2024). Precision spectral tuning, optimized light distribution, and adaptive photoperiod management can enhance resource-use efficiency, improve crop quality, and maximize productivity while minimizing energy expenditure.

## Leveraging plant-microbe interactions to enhance crop performance in controlled environments

Integrating plant-microbe interactions in CEA presents a promising opportunity for improving nutrient efficiency, enhancing stress resilience, and optimizing plant health. Beneficial microbial inoculants, including fungi and bacteria, can promote plant growth through nutrient solubilization (Shahwar et al., 2023), root architecture modification (Galindo-Castañeda et al., 2022), and systemic resistance induction (Elnahal et al., 2022). However, the success of microbial applications depends on environmental conditions such as light intensity, nutrient availability, and plant species specificity.

Tan et al. examined *Trichoderma harzianum* in *Nicotiana benthamiana* under different light conditions, revealing that microbial symbiosis is a dynamic process influenced by environmental cues. Under high-light conditions, *Trichoderma* enhanced plant growth and nutrient uptake; however, under low-light conditions, *Trichoderma* became parasitic, hindering plant growth and phosphorus assimilation. These findings emphasize the importance of maintaining optimal lighting conditions to foster mutualistic relationships between plants and microbes.

Microbial interactions also play a key role in nutrient cycling and leachate management in hydroponic systems. *Trichoderma* has been shown to improve phosphorus solubilization, which aligns with the findings by Westmoreland and Bugbee on phosphorus leaching in *Cannabis sativa* (see also Hershkowitz et al., 2025). Leveraging microbial solutions for phosphorus recycling could enhance the sustainability of closed-loop hydroponic systems.

## Future directions: innovation and sustainability in controlled environment agriculture

The future of vertical farming lies in the continued refinement of multidisciplinary approaches that integrate plant biology, lighting optimization, nutrient recycling, microbial interactions, and automated environmental control. While current advancements have demonstrated the feasibility of high-efficiency indoor agriculture, several challenges remain in achieving widespread scalability and sustainability. The integration of artificial intelligence and machine learning for real-time climate control, precision fertigation, and automated plant phenotyping represents one of the most promising directions for improving system efficiency and reducing resource waste. Data-driven models combined with process-based models, as described by Abedi et al., that predict plant growth responses to dynamic environmental variables could enable farms to fine-tune lighting, nutrient delivery, and CO_2_ supplementation in ways that maximize yield while minimizing energy consumption.

One area of interest for future research is the in-depth exploration of plant-microbe interactions in controlled environments and hydroponic cultivation. While studies have shown that beneficial microbes, such as *Trichoderma harzianum*, can enhance nutrient uptake and stress resilience, their efficacy is often contingent upon environmental conditions. In some cases – as described by Tan et al. – the mutualism can become parasitism. A more detailed understanding of the response of these interactions to varying light spectra, humidity, and nutrient availability will be essential to optimizing microbial applications for vertical farming.

Additionally, environmental sustainability will be a key driver in the future of controlled environment agriculture. Future research should focus on drastic improvements in energy (light) use efficiency. Furthermore, integrating renewable energy sources such as solar or geothermal power into vertical farming operations could help mitigate the high energy costs associated with artificial lighting and climate control (Kaiser et al., 2024). Closed-loop water and nutrient recycling systems will also play a crucial role in minimizing waste and improving overall efficiency. Advances in real-time sensor technology will allow for precise monitoring of plant physiological responses, enabling a level of control that enhances productivity while reducing environmental impact.

## Conclusion

This Research Topic of *Frontiers in Plant Science* showcases cutting-edge research that advances our understanding of plant biology in indoor vertical farming systems. It highlights that vertical farming research is not limited to plants grown in vertical farms but extends to the knowledge gained from plants cultivated under artificial conditions. Furthermore, it underscores the inherent complexity of integrating and interpreting multiple parameters—including light, nutrients, biophysics, and microbiome interactions. This complexity makes vertical farming challenging to operate but presents an unprecedented opportunity to optimize our food system by maximizing resource efficiency and crop productivity.

By integrating multidisciplinary research, vertical farming can evolve into a truly sustainable, high-efficiency food production system. Advancements in nutrient optimization, light management, and plant-microbe interactions provide a foundation for future innovations. The incorporation of real-time monitoring technologies, precision fertigation, and adaptive climate control will be essential to driving the next generation of controlled environment agriculture and ensuring food security in a changing global landscape.
